# Use of open‐source software and photogrammetry in the fabrication of 3D‐printed custom trays for edentulous patients: A technical guide

**DOI:** 10.1111/jopr.70035

**Published:** 2025-09-20

**Authors:** João Victor Cunha Cordeiro, Lauren Bohner, Fernanda Pretto Zatt, Artur Ferronato Soto, Henrique Souza dos Santos, Maria Eduarda Broering da Silva, Maurício Malheiros Badaró, Ricardo Armini Caldas

**Affiliations:** ^1^ Department of Prosthodontics and Periodontology Piracicaba Dental School University of Campinas (UNICAMP) Piracicaba São Paulo Brazil; ^2^ Department of Dentistry Federal University of Santa Catarina (UFSC) Florianopolis Santa Catarina Brazil

**Keywords:** computing methodologies, dentures, digital technology, photogrammetry, printing, three‐dimensional

## Abstract

A technique is described to obtain a 3D‐printed custom tray for complete dentures through open‐source, free software and additive manufacturing. This approach enables the production of custom trays in a cost‐effective and optimized manner. A three‐dimensional (3D) digital cast is obtained by photogrammetry and later used to design a digital custom tray. Then, the digital custom tray is manufactured by a 3D printer to be used in clinical procedures. This technique is a clean, rapid, and low‐cost alternative to the traditional technique.

Custom trays play an important role in the manufacturing of complete dentures, as the quality of the functional impression forms the foundation for denture retention and stability.^1^ A satisfactory functional impression must capture precise anatomical landmarks of the edentulous jaws, ensure proper extension, and reflect the functional morphology of the surrounding tissues.^2^

Traditionally, custom trays are fabricated using auto‐polymerizing acrylic resin based on an anatomical impression. However, this method can be time‐consuming, as it requires detailed laboratory procedures and adjustments in the patient's mouth.^3^ These factors may result in errors, as the nonuniform relief distances from the functional impression to the custom tray due to resin deformation.^4^

With the advent of additive manufacturing technologies such as three‐dimensional (3D) printing, traditional oral rehabilitation methods have significantly improved. 3D‐printed custom trays allow for more controlled relief distances and offer a cost‐effective workflow, compared to other techniques.^5^ In this way, fused deposition modeling (FDM) printers have gained popularity due to their effectiveness in the production of prosthetic components with satisfactory resolution, dimensional stability, ease of handling, and fast operation, using biocompatible materials, such as polylactic acid (PLA).^6^, ^7^, ^8^

Despite these advancements, replacing conventional dental impressions with digital impressions requires high‐cost technologies (e.g., intraoral scanners), which may not be accessible to general dental practitioners. For this reason, the development of low‐cost alternative techniques like photogrammetry, may be required.^9^, ^10^ In terms of accessibility, photogrammetry used to demand high‐cost equipment and workstations for 3D model reconstruction. However, due to advances in computer processing, even smartphone cameras can now help produce reliable and accurate 3D models.^9^, ^11^ Although the fabrication of custom trays via additive manufacturing has already been validated with clinically acceptable accuracy,^12^ no previous study has proposed a workflow that combines smartphone‐based photogrammetry with open‐source software for the design and manufacturing of custom trays, particularly for edentulous patients.

This report presents an innovative and cost‐effective technique for fabricating 3D‐printed custom trays for complete dentures through photogrammetry. The workflow employs free, open source software and additive manufacturing. The technique is simple and offers an accessible solution for dental professionals.

## TECHNIQUE

The reported technique was approved by the institutional ethics committee (CAAE: 28171420.8.0000.0121). A 65 year‐ old patient went to the dental clinic unsatisfied with her complete denture. A detailed anamnesis and clinical examination confirmed the need for replacement of the old dentures. The following steps were taken to manufacture the custom tray.

**FIGURE 1:**
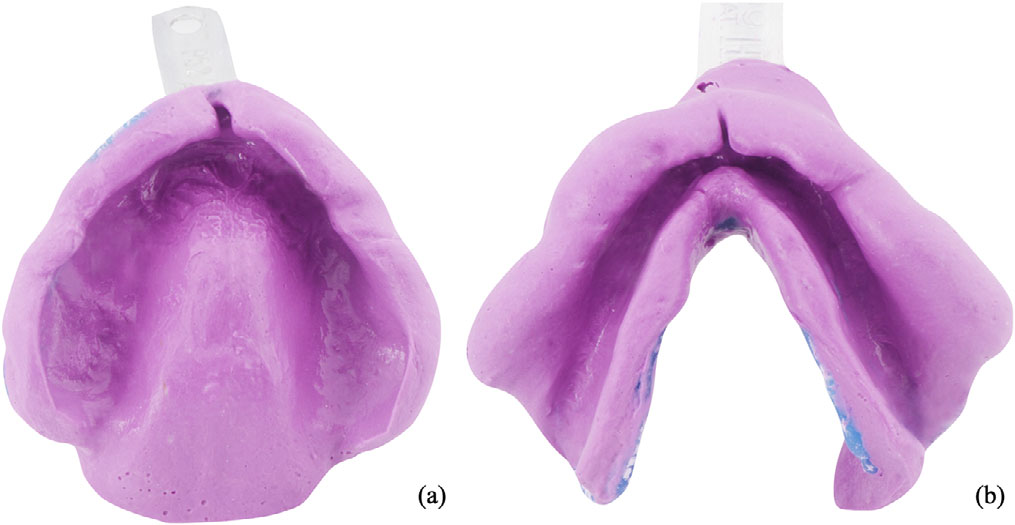
Preliminary impression with irreversible hydrocolloid material (Hydrogum 5; Zhermack GmbH). (a) Upper jaw; (b) Lower jaw.

**FIGURE 2:**
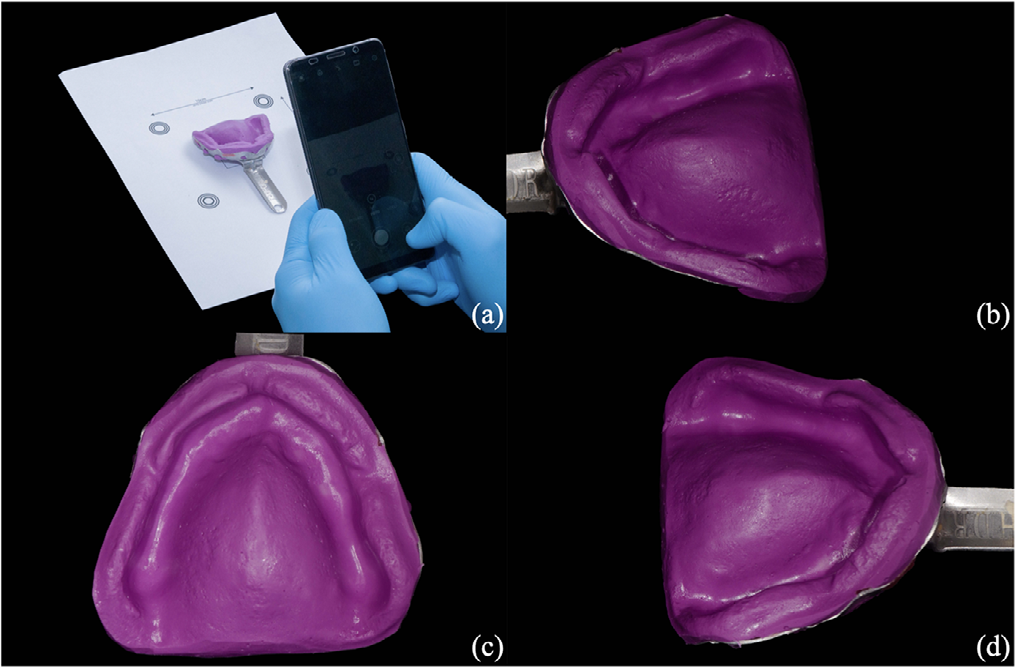
Scanning protocol. (a) Positioning of the smartphone in relation to the impression. (b) Left side of the impression. (c) Occlusal view of the impression. (d) Right side of the impression.

**FIGURE 3:**
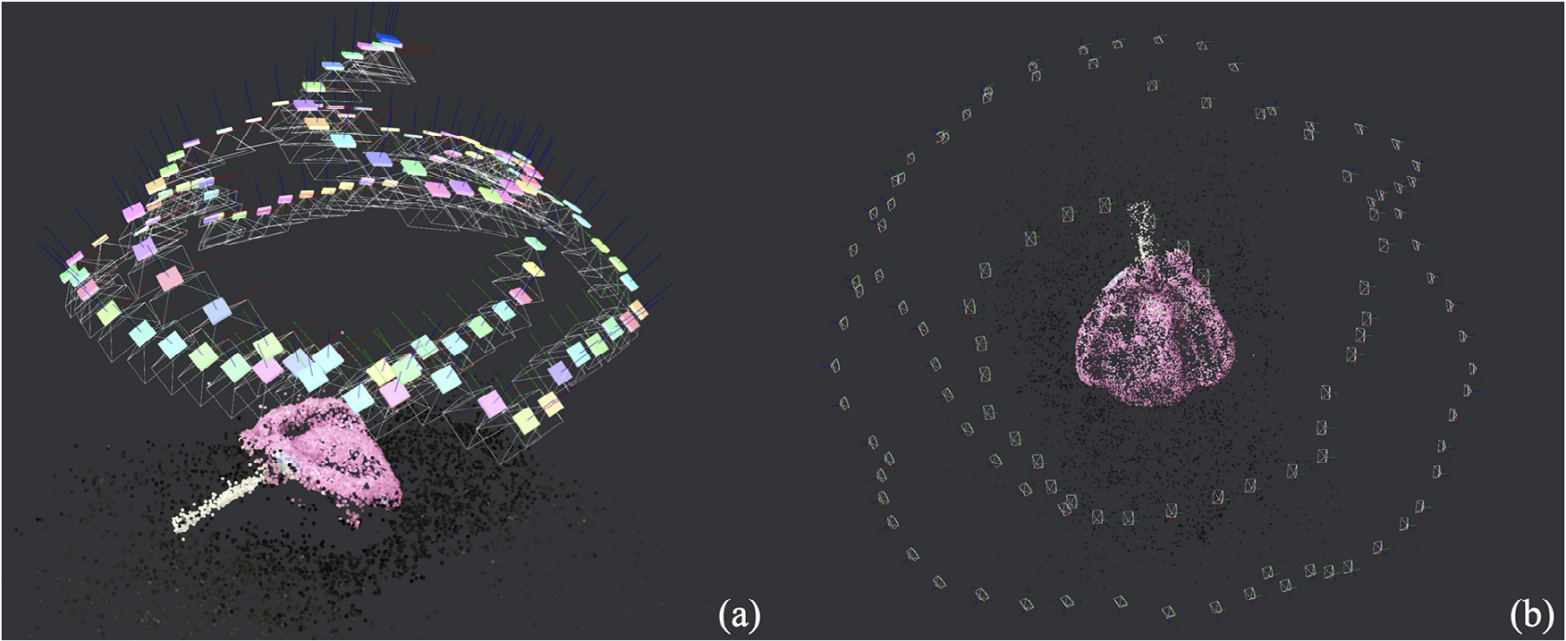
(a) Isometric view of the camera recordings surrounding the preliminary impression; (b) top view of the camera recordings surrounding the preliminary impression.

**FIGURE 4:**
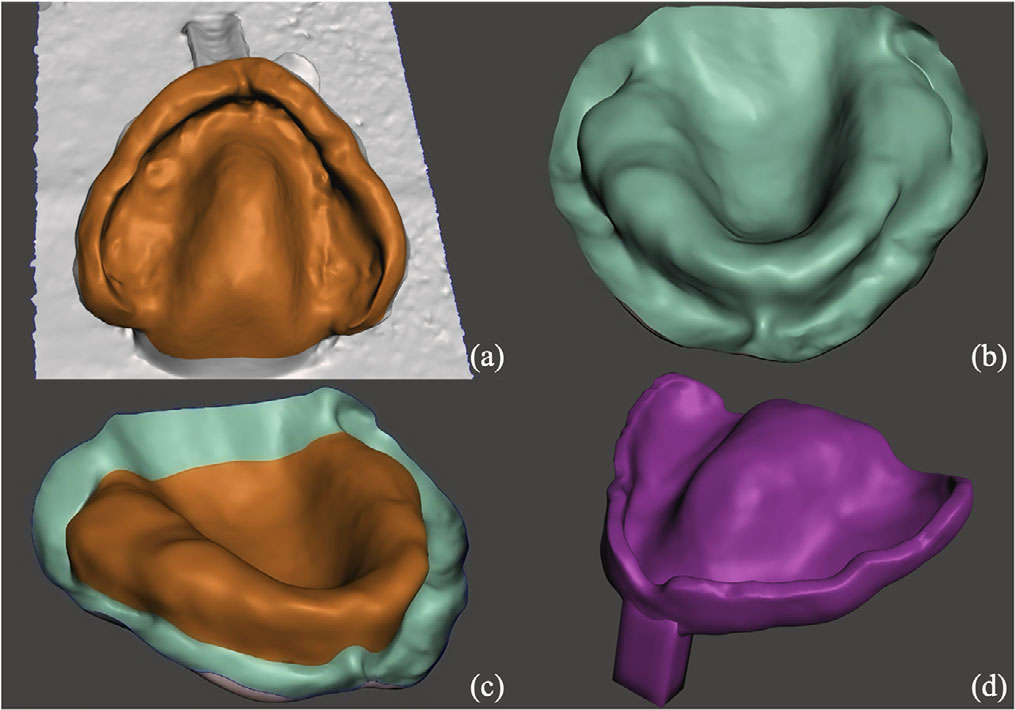
3D model based on photogrammetry of the preliminary cast. (a) Trim unwanted areas of the model; (b) invert normal to obtain preliminary cast. (c) delimit the denture supporting area, and with the supporting area selected, extrude 3 mm and then design a handle for the tray; (d) final model of the custom tray.

1. Make a preliminary impression using an irreversible hydrocolloid material (Hydrogum 5; Zhermack) (Figure 1a and b).
2. Print a CCTag sheet (available at https://github.com/ricardocaldas/cctagpipeline), which is required for the calibration of the digital model. Place the preliminary impression over the sheet (Figure 2a), and record a 30‐second video using either a digital camera or a smartphone (Huawei Mate 10 Pro; Huawei). Keep a minimal resolution of at least 1080p and 30fps. Move the device slowly around the preliminary impression, ensuring a distance of 30 cm to the sheet^13^ (Figure 2b, c, and d).
3. In Photogrammetry Software (Alicevision Meshroom 2021.1.0; AliceVision), extract the frames from the video to import them as pictures (Figure 3a and b). Use the Photogrammetry Pipeline of Alicevicison Meshroom with the CCTag feature to create the 3D model in the correct scale. Predefined pipelines with CCTag and KeyframeSelection are presented at https://github.com/ricardocaldas/cctagpipeline.
4. Based on the preliminary impression, design the 3D model using the tools “trim unwanted areas” (Figure 4a) and “invert normal” (Figure 4b) of the modeling software (Autodesk Meshmixer 3.5; Autodesk).
5. Design the custom tray as follows: define the denture supporting area (Figure 4c), ensuring a distance of 1–2 mm from the tray border to the gingivolabial and gingivobuccal sulcus. After, extrude the selected area to reach a thickness of 3 mm on the main area and 3–4 mm on the borders. Additionally, design a handle for the tray, ensuring an angle of 45° to the tray (Figure 4d, 5a and b).^14^ Refine the internal surface of the tray using the Smooth function in Meshmixer, applied to the photogrammetry‐based mesh. This procedure reduces surface irregularities and sharp edges, generating a functional biological relief that facilitates the proper flow of the impression material and prevents excessive tissue compression. This refinement allows the fabrication of a tray that promotes a regular impression with consistent material thickness across all areas. Print the designed tray using a FDM printer with PLA and a layer 0.2 mm high (Figure 6a and b).
6. Make the clinical adjustments intraorally with burs (Figure 7a and b). Perform the functional impression using an elastomeric material. The roughness of the custom tray creates mechanical retention for the impression material, which eliminates the use of adhesive for the tray. In this phase, avoid using thermoplastic impression materials or low‐fusion impression compounds, as they may cause tray distortion (Figure 8a and b). Perform the next steps of manufacturing a conventional complete denture up to its insertion (Figure 9).

**FIGURE 5:**
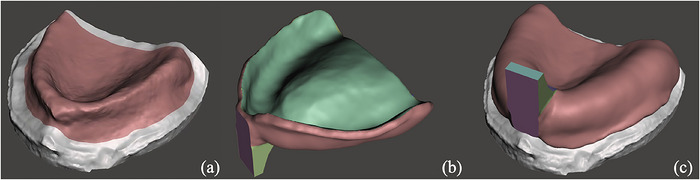
3D model based on photogrammetry of preliminary cast. (a) Preliminary cast with supporting area selected; (b) with supporting area selected, extrude 3 mm and then design a handle for the tray; (c) custom tray placed over preliminary cast.

**FIGURE 6:**
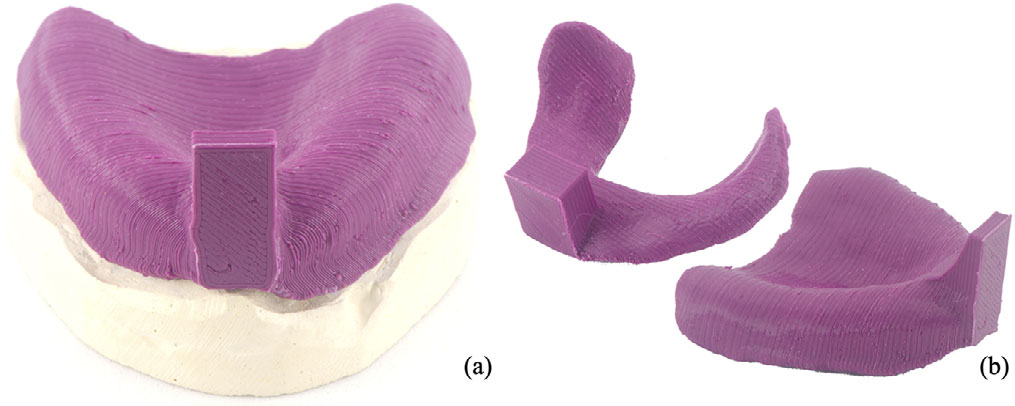
3D‐printed custom trays. (a) Custom tray on preliminary cast attesting the adaptation; (b) upper and lower custom trays.

**FIGURE 7:**
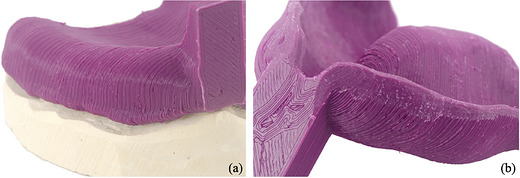
Adjustments before functional impression. (a) Checking adjustments at the preliminary cast; (b) final adjustments.

**FIGURE 8:**
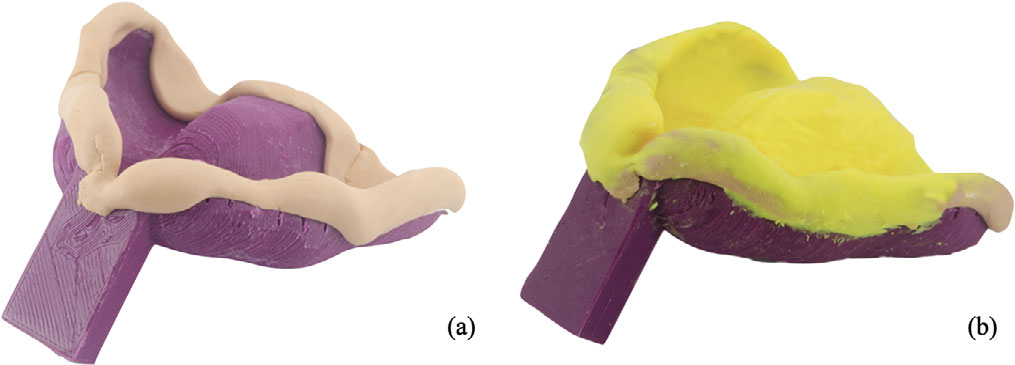
Two‐step functional impression with addition‐type silicone material. (a) First step with dense material; (b) second step with flowable material.

**FIGURE 9:**
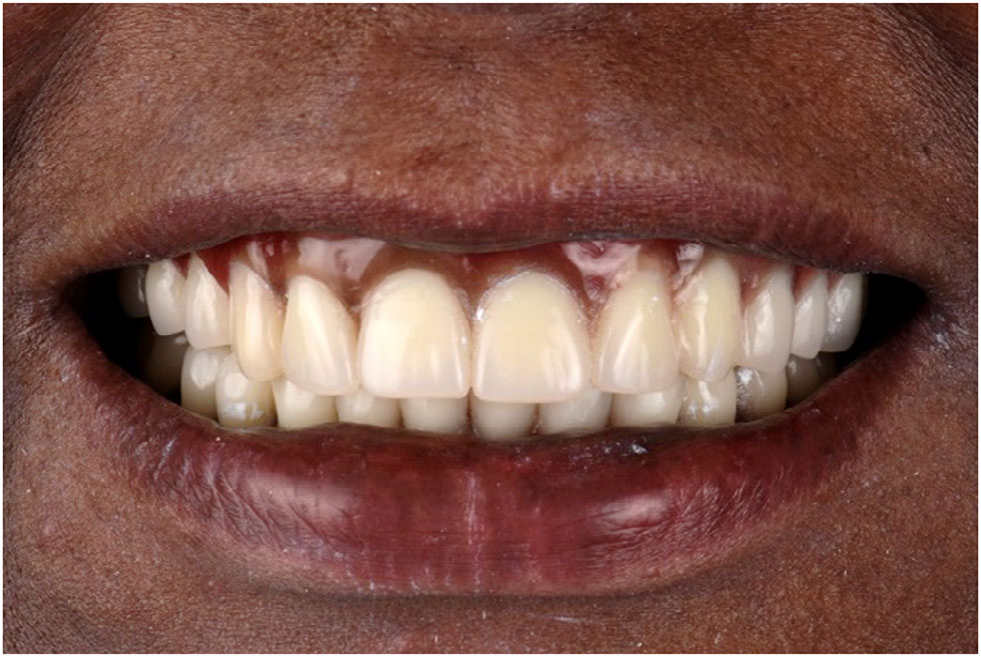
Inserted conventional complete denture.

## DISCUSSION

Digital workflows are increasingly integrated into clinical practice in dentistry, enabling accurate and time‐efficient procedures. One of the main advantages of 3D scanning systems is their ability to capture highly accurate digital models, which significantly improve the quality and predictability of treatments.^15^ Photogrammetry offers a more accessible solution by utilizing devices like smartphones to capture digital models. This approach reduces costs while maintaining practicality and flexibility for clinicians. ^16^

Previous studies demonstrated that photogrammetry can achieve levels of accuracy comparable to those of traditional scanning methods.^17^, ^18^, ^19^ Zotti et al.^19^ evaluated the accuracy of digital dental models created using photogrammetry in comparison with extraoral and intraoral scanners. The study showed no significant differences in accuracy among the methods, and photogrammetry showed a high level of agreement with plaster models.

Their findings highlighted that photogrammetry offers a reliable, cost‐effective alternative for digitizing dental models. In this sense, smartphones could provide low‐cost and easily accessible tools for 3D scanning and modeling. Nonetheless, the accuracy of smartphones in capturing precise details is still lower compared to dedicated scanning devices.^16^ Although the use of smartphone‐based photogrammetry has shown promising results for digitizing dentate dental models,^20^ its applicability to edentulous arches remains uncertain. The smooth and geometrically less distinct morphology of edentulous surfaces poses a unique challenge for feature detection and 3D reconstruction algorithms. As a result, the accuracy and reliability of photogrammetry in capturing clinically relevant anatomical landmarks in edentulous patients has not yet been established. This gap in the prosthodontic literature underscores the need to explore whether low‐cost and accessible digital tools can be successfully integrated into complete denture workflows, particularly during the preliminary impression phase

A custom tray made with an FDM 3D printer using PLA has advantages over self‐cure resin, promoting uniformly distributed reliefs that allow for functional molding. In addition, the rough surface of custom FDM‐printed PLA trays can improve the retention of the impression material, simplifying the technique and eliminating the need for tray adhesive.^5^ Beyond these benefits, PLA also offers biocompatibility^21^ and cost‐efficiency.

The main limitation of this technique is the contra‐indication of using thermoplastic material for impressions, due to the low fusion point of PLA at 60°C. Furthermore, photogrammetry requires a learning curve, since it comprises uncommon steps for a dental clinical procedure, while high‐end scanning technology produces a digital model in a more user‐friendly workflow.^22^ Nevertheless, a standardized protocol can overcome this limitation, and the technique can be replicated either by clinicians or by dental technicians.

Further research is necessary to assess the quality of digital models obtained through photogrammetry compared to conventional methods. Additionally, research into the fit of the stackable structure and improvement in the custom occlusion rim is required for further development of digitally fabricated dentures.

## SUMMARY

A method of fabricating a CAD‐CAM custom record tray for complete dentures using photogrammetry and 3D printing has been described. This technique enables the cost‐effective use of digital resources for the fabrication of complete dentures and enhances the effectiveness of the custom record tray. Nevertheless, further studies comparing this technique with conventional methods are essential to assess its accuracy.

## CONFLICT OF INTEREST STATEMENT

The authors declare no conflicts of interest.

## Supporting information




Supporting Information
