# Supplementary material for: Use of open‐source software and photogrammetry in the fabrication of 3D‐printed custom trays for edentulous patients: A technical guide
Source: J Prosthodont. 2025 Sep 20;35(7):1164–8. doi: 10.1111/jopr.70035 (PMC13456456; doi:10.1111/jopr.70035)
Supplement: Supplementary file 1 [file JOPR-35-1164-s001.docx]

**WORKFLOW FOR 3D MODEL GENERATION AND PREPARATION FOR 3D PRINTING**

# 1. Video Capture and Frame Extraction

The photogrammetry process employed in this workflow is based on video capture to generate the set of images required for accurate 3D reconstruction. Videos are recorded using a smartphone camera with a minimum resolution of 1080p (1920 × 1080 pixels) and 30 frames per second (fps), while maintaining a constant distance of approximately 30 cm from the object. The video acquisition is performed through 360-degree circumferential passes at varying heights and angles (low, mid, and high), ensuring comprehensive coverage of all anatomical surfaces of the object.

A CCTag calibration sheet, along with the corresponding usage tutorial (available at: https://github.com/ricardocaldas/cctagpipeline) in A4 format, was placed beneath the preliminary impression to enable dimensional calibration of the digital model during the subsequent 3D modeling process. This procedure ensures that the 3D reconstruction is accurately scaled, allowing the digital model to precisely correspond to the real-world dimensions of the object.

# 2. Frame Extraction from Video

The recorded video is subsequently imported into frame extraction software. The following free tools are recommended for this step:

- VLC Media Player (available at: https://www.videolan.org/) (utilizing frame extraction function)

A frame extraction rate of 1 frame per second is typically employed, although manual selection of sharp, blur-free frames is recommended when necessary. This process typically yields 80 to 120 high-quality images, which is sufficient to achieve optimal reconstruction fidelity.

# 3. 3D Reconstruction in Meshroom (AliceVision)

Open Meshroom (version 2023.3.0) (available at: https://alicevision.org/#meshroom) and create a new project directory. Import the extracted images by dragging them into the “Images” panel. Confirm that all images are properly recognized by the software, with camera metadata correctly identified (e.g., focal length). The photogrammetry pipeline is initiated by clicking the “Start” button, which processes the following sequential steps:

- Feature Extraction
- Image Matching
- Structure from Motion (camera alignment and sparse point cloud generation)
- Depth Map Computation
- Depth Map Fusion (dense point cloud)
- Mesh Generation
- Mesh Filtering
- Texturing (optional; not required for 3D printing)

The CCTag calibration sheet placed beneath the preliminary impression is detected during the photogrammetry pipeline to enable metric calibration of the 3D model. The CCTag markers are recognized automatically during the Structure from Motion step and embedded into the reconstruction process, ensuring that the generated mesh preserves real-world scale and proportions. This step eliminates the need for manual scaling adjustments during the modeling phase and guarantees that the digital model accurately corresponds to the physical dimensions of the object.

# 4. Exporting the 3D Model from Meshroom

Upon completion of the reconstruction process, the output mesh is automatically saved in the directory: \Your_Project\MeshroomCache\Texturing\XXXXXXX\. The 3D model is exported in OBJ format, along with auxiliary files such as MTL and PNG if texturing is applied. However, for 3D printing purposes, only the geometric data contained in the OBJ file is required, as the texture files are not relevant for manufacturing.

# 5. Post-Processing in Meshmixer

Open Autodesk Meshmixer (version 3.5.0) (available at: https://apps.autodesk.com/FUSION/en/Detail/Index?id=4108920185261935100&appLang=en&os=Win64). Perform the following post-processing steps:

- Import the OBJ file via File - Import.
- Inspection and Repair: Navigate to Analysis - Inspector to automatically detect and correct mesh errors, such as holes, non-manifold edges, and disconnected vertices.
- Extrusion: Use Edit - Extrude to increase wall thickness if additional mechanical strength is required for 3D printing.
- Smoothing: Apply Sculpt - Smooth to manually refine specific surface areas, reducing mesh roughness and improving the overall surface finish.
- Additionally, use Select - Modify - Smooth Boundary to soften and regularize the edges of selected regions, particularly after operations like Plane Cut, ensuring smooth boundary transitions.
- Scaling Verification: Confirm the dimensional accuracy under Analysis - Units/Dimensions and apply scaling adjustments if necessary to ensure correspondence with the physical model.
- Plane Cut: Apply Edit - Plane Cut to remove unnecessary geometry, including background artifacts and scanning noise.
- Export the finalized mesh in STL format via File - Export - STL Binary (*.stl) for subsequent 3D printing.

# 6. Slicing for 3D Printing

Open a slicing software compatible with FDM 3D printing, such as Orca Slicer (available at: https://orca-slicer.com) or Cura Slicer (available at: https://curaslicer.com/).
Import the STL file via File - Open or by drag-and-drop.

Apply the following recommended slicing parameters for fabricating the custom tray:

- Layer height: 0.2 mm
- Wall line count: 3 perimeters
- Infill density: 20% to 30% (grid or cubic pattern)
- Top and bottom layers: 4 to 5
- Supports: Enable if necessary, particularly for handles or overhang regions
- Build plate adhesion: Skirt or brim, depending on the tray geometry

Orient the tray flat against the build plate using the Lay Flat function to optimize adhesion, reduce warping, and ensure dimensional stability during printing.

The slicing process generates a G-code file, which can be saved to an SD card, USB drive, or transferred directly to the 3D printer, depending on the hardware configuration.

# 7. Summary of Digital Workflow:

Video Capture (MP4) → Image Extraction (JPG/PNG) → 3D Reconstruction in Meshroom (OBJ) → Mesh Processing in Meshmixer (STL) → Slicing Software (G-code) → 3D Printed Custom Tray
